# The inhaled phosphodiesterase 4 inhibitor GSK256066 reduces allergen challenge responses in asthma

**DOI:** 10.1186/1465-9921-11-26

**Published:** 2010-03-01

**Authors:** Dave Singh, Frank Petavy, Alex J Macdonald, Aili L Lazaar, Brian J O'Connor

**Affiliations:** 1 The University of Manchester, Manchester Academic Health Science Centre, University Hospital Of South Manchester NHS Foundation Trust, Manchester M23 9LT, UK; 2 Medicines Evaluation Unit, Langley Building, Southmoor Road, Manchester M23 9QZ, UK; 3 GlaxoSmithKline, Stevenage, UK; 4 GlaxoSmithKline, King Of Prussia, PA, USA; 5 Department of Asthma, Allergy and Respiratory Science Guy's, King's and St Thomas' School of Medicine at King's College Hospital, Bessemer Road, London SE5 9PJ, UK

## Abstract

GSK256066 is a selective phosphodiesterase 4 inhibitor that can be given by inhalation, minimising the potential for side effects. We evaluated the effects of GSK256066 on airway responses to allergen challenge in mild asthmatics.

**Methods:**

In a randomised, double blind, cross-over study, 24 steroid naive atopic asthmatics with both early (EAR) and late (LAR) responses to inhaled allergen received inhaled GSK256066 87.5 mcg once per day and placebo for 7 days, followed by allergen challenge. Methacholine reactivity was measured 24 h post-allergen. Plasma pharmacokinetics were measured. The primary endpoint was the effect on LAR.

**Results:**

GSK256066 significantly reduced the LAR, attenuating the fall in minimum and weighted mean FEV1 by 26.2% (p = 0.007) and 34.3% (p = 0.005) respectively compared to placebo. GSK256066 significantly reduced the EAR, inhibiting the fall in minimum and weighted mean FEV1 by 40.9% (p = 0.014) and 57.2% (p = 0.014) respectively compared to placebo. There was no effect on pre-allergen FEV1 or methacholine reactivity post allergen. GSK256066 was well tolerated, with low systemic exposure; plasma levels were not measurable after 4 hours in the majority of subjects.

**Conclusions:**

GSK256066 demonstrated a protective effect on the EAR and LAR. This is the first inhaled PDE4 inhibitor to show therapeutic potential in asthma.

**Trial Registration:**

This study is registered on clinicaltrials.gov NCT00380354

## Introduction

Inhaled corticosteroids are the cornerstone of anti-inflammatory treatment in asthma [1]. However, many patients remain symptomatic despite high doses of inhaled corticosteroids, even when combined with long acting beta agonists [2,3]. New asthma treatments targeting inflammation are needed.

Adenosine monophosphate (cAMP) and cyclic guanosine monophosphate (cGMP) cause smooth muscle relaxation and regulate immune cell function [4]. These intracellular signalling molecules are inactivated by the phosphodiesterase (PDE) family of metallophosphohydrolases, which can lead to smooth muscle contraction and increased immune cell activation [4,5]. Therefore, the non-selective oral PDE inhibitor theophylline has been used as a treatment for asthma for many years. However, it has a low therapeutic index due to limited potency and a poor side effect profile [6,7]. The PDE4 subfamily are highly expressed on inflammatory cells such as eosinophils, lymphocytes, macrophages and neutrophils [5,8], so selective PDE4 inhibitors have recently been developed with the aim of improving the therapeutic index. Animal models have shown this approach to be highly effective in reducing allergen induced inflammation [9,10]. Clinical studies have shown efficacy for orally administered PDE4 selective inhibitors on relevant asthma endpoints such as inhibition of allergen challenge [11,12] and exercise induced bronchoconstriction [13], as well as improvements in lung function [14]. However, the tolerability of these orally administered drugs is still limited by side effects such as gastro-intestinal symptoms [15-17].

The delivery of a selective and potent PDE4 inhibitor by inhalation may improve the therapeutic index by limiting systemic exposure and delivering the drug directly to the target organ to increase therapeutic effects. GSK256066 (6-({3- [(dimethylamino) carbonyl]phenyl}sulfonyl)-8-methyl-4-{ [3-methyloxy)phenyl]amino}-3-quinolinecarboxamide) is a PDE4 inhibitor that can be delivered by inhalation. This compound is a very high affinity, slow- and tight-binding inhibitor of PDE4 that is highly selective for PDE4 over other PDEs such as 1, 2, 3, 5, 6 and 7, and shows efficacy in animal models of pulmonary inflammation [18].

The aim of this study was to investigate the effects of selective inhibition of PDE4 with GSK256066 delivered by inhalation in the experimental allergen challenge model of allergic asthma. We performed a double blind, placebo controlled, crossover study in steroid naïve asthma patients to assess the effectiveness of GSK256066. We also measured systemic exposure to GSK256066.

## Methods

### Subjects

24 steroid naïve patients with physician diagnosed asthma for at least 6 months were recruited - the demography of the patients is shown in table 1. Subjects were required to be aged 18 to 55 years and non-smokers for at least 6 months with less than a 10 pack year history. At screening patients were required to have a forced expiratory volume in 1 second (FEV_1_) > 75% predicted, have a positive skin test to either house dust mite, grass pollen or cat allergen, and to demonstrate both an early and late asthmatic reaction to one of these allergens when inhaled. Subjects we also required to have haematology, biochemistry and creatinine clearance values within the normal ranges. All patients provided written informed consent. The study was approved by the local research ethics committee.

**Table 1:** Subject Demography.

Variable	Value
Age/years	31 (20 - 46)

Gender (Male/Female)	13/11

FEV1 % predicted	90.1 (71.3 - 111.8)

Allergen used for bronchial challenge	14 dust mite, 5 cat dander, 5 grass mix

Mean (range) shown for age and FEV1.

### Study Design

**Figure 1:**
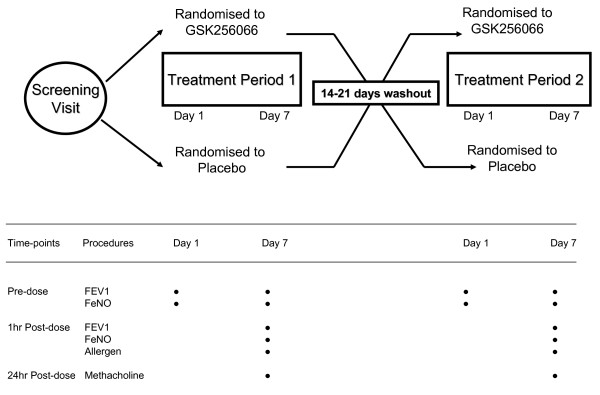
**Flow chart showing study design**. FeNO denotes exhaled nitric oxide. Allergen = inhaled allergen challenge. Methacholine = inhaled methacholine challenge.

This was a two centre, double-blind, randomised, placebo controlled, cross-over study. Eligible subjects were randomised to receive GSK256066 87.5 μg or matching placebo using an Accuhaler™ once daily for 7 days - see Fig 1. The washout period was 14 - 21 days between treatment periods. Dosing was performed under supervision at the sites on Day 1 and Day 7. On days 2-6 subjects were instructed to take the study medication at the same time of day, and were required to complete a diary card to document the time that medication was taken. Heart rate, blood pressure, ECGs, FEV_1 _and exhaled nitric oxide (FeNO) were measured pre-dose on days 1 and 7, and at 1 hr post-dose. On day 7, an inhaled allergen challenge was subsequently performed after the 1 hour post-dose FEV_1 _and FeNO measurements. Methacholine challenge was then performed at 24 hours post allergen challenge. Adverse events and beta agonist use were monitored throughout the study with the aid of diary cards.

### Allergen and Methacholine Challenges

Bronchial challenges were performed as we have previously described [19] using a Mefar Dosimeter (Mefar-Bologna). Allergen for skin prick tests (Soluprick SQ, Alk Abelló (UK) Ltd) was stored at 4°C; each subject was assessed for sensitivity to house dust mite, cat, grass pollen, and positive and negative controls. The allergen for inhalation was selected according to the largest skin test wheal (positive >3 mm) and clinical history. Fresh solutions of allergen were made up in 0.9% saline in doubling concentrations from 250 SQ-U/ml to 32 000 SQ-U/ml. At screening, incremental doses of allergen were inhaled [19] until an early asthmatic response (EAR) was observed, defined as a fall in FEV1 of ≥ 20% from the post saline value, on at least one occasion, between 5 and 30 minutes after the final concentration of allergen. The late asthmatic response (LAR) was defined as a fall in FEV1 of ≥ 15% from the post saline value, on at least three occasions, two of which must be consecutive, between 4 and 10 hours after the final concentration of allergen. During the treatment periods, the total dose of allergen required to cause an EAR and LAR was administered as a single bolus dose.

Subjects were administered doubling concentrations of methacholine from 0.03125 to 32 mg/ml until a ≥ 20% fall in FEV_1 _was achieved or the highest concentration of methacholine was administered. The provocative concentration required to reduce the FEV_1 _by 20% of the post-saline baseline value (PC_20_) was derived by linear interpolation between the lowest concentration that caused a >20% fall and the preceding concentration. If the FEV_1 _did not fall by more than 20% following the highest concentration then the PC20 was set to the highest concentration given in the challenge. If the FEV_1 _fell by more than 20% following the first concentration the PC20 was derived as [20× lowest concentration]/[% fall following lowest concentration].

### FeNO

FeNO was measured using the Ecomedics AG analyser CLD 88 at a flow of 50 ml/s. Three acceptable readings were recorded from each subject and the mean was used for analysis.

### Pharmacokinetic Sampling and Bioanalytical Method

On days 1 and 7, blood samples were collected at pre-dose, 10 min, 30 min, 45 min and 1, 2, 3, 4, 6, 8, 10, 11, 12 and 24 hrs post dose for measurement of the levels of GSK256066 in plasma. The active metabolite GSK614917, which is 1.7 fold less potent than the parent compound, was also measured. These pharmacokinetic analyses were performed by protein precipitation, followed by HPLC/MS/MS. The lower limit of quantification (LLOQ) for GSK256066 and GSK614917 was 5 pg/mL, with an upper limit of quantification of 2000 pg/mL

### Statistics

Minimum LAR was derived as the minimum FEV1 value over 4-10 hrs post allergen challenge. Minimum EAR was derived as the minimum FEV1 over 0-2 hrs post allergen challenge. Weighted mean LAR and EAR endpoints were derived by calculating the AUC over the relevant time interval using the linear trapezoidal rule and dividing by the time interval. The sample size was based on a power calculation using our previous allergen challenge data [19]; in order to detect a 50% attenuation of the minimum LAR, with 90% power at the two-sided 5% significance level, 23 evaluable were required. Statistical analysis was performed on each of the absolute change from baseline LAR and EAR endpoints to compare GSK256066 with placebo. A mixed effects model was fitted with the factors treatment, period and Day 7 post-saline FEV1 as fixed effects and subject as a random effect. Absolute change from post-saline FEV1 data over planned relative time were obtained from a repeated measures statistical analysis, adjusting for the terms of period, treatment group, period-level post-saline baseline, subject-level post-saline baseline, planned relative time, treatment group by planned relative time interaction and period-level post-saline baseline by planned relative time interaction as fixed effects and subject as a random effect. Day 7 FEV1 data (pre-dose and 1 h post-dose) were also analysed using a mixed effects model, adjusting for the fixed effects treatment, period and Day 1 pre-dose FEV1 and the random effect subject. Statistical analysis was performed on the log_2_-transformed values of the provocative concentration of methacholine required to produce a 20% reduction in FEV1 (PC20) to compare GSK256066 with placebo. A mixed effects model was fitted with the factors treatment and period treated as fixed effects and subject as a random effect. FeNO change from baseline ratio at all time points were analysed following a log_e_-transformation to compare GSK256066 with placebo. A mixed effects model was fitted with the fixed effects period, treatment group, subject-level log_e_-transformed baseline, period-level log_e_-transformed baseline, planned relative time, treatment group by planned relative time interaction term and period-level log_e_-transformed baseline by planned relative time interaction term and the random effect subject.

Values for the following pharmacokinetic were estimated directly from raw plasma concentration data: maximum plasma concentration (Cmax), time of maximum observed concentration (Tmax), and time of the last observable concentration (Tlast). Area under the plasma concentration-time curve from time zero to Tlast (AUC (0-t)) was estimated for subjects with at least 3 consecutive observable concentration values with the log up/linear down trapezoidal method using Winnonlin professional version 5.2 (Pharsight Corporation, Cary, NC, USA).

## Results

**Figure 2:**
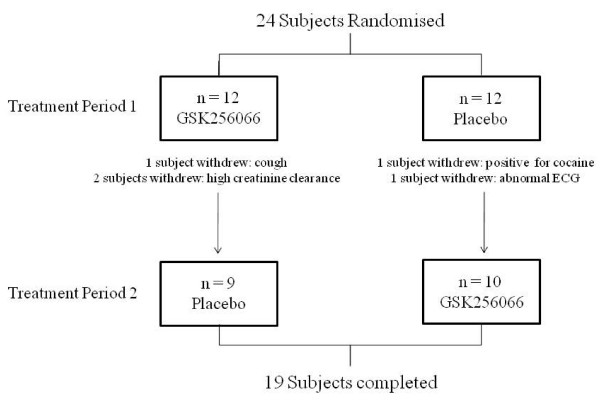
**Flow chart showing withdrawal of subjects during the study**.

Of the 24 subjects randomised, 19 completed the study (see Fig 2). One subject developed cough and wheeze after inhalation of 3 doses of GSK256066 which resolved within 24 hrs. Two subjects had evidence of high creatinine clearance during the study; one after placebo, and one after three doses of GSK256066. Two withdrawals occurred during placebo treatment (the subjects did not receive GSK256066); one subject tested positive for cocaine, and one subject had abnormal ECG changes.

### Allergen Challenge

**Figure 3:**
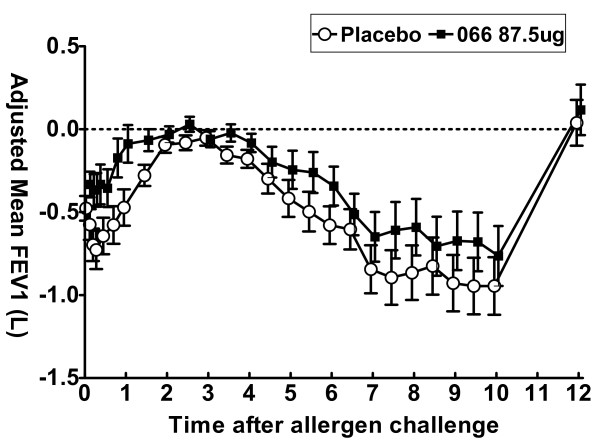
**Early and late asthmatic response to inhaled allergen challenge after 7 days treatment with either GSK256066 or placebo**. Means and 95% confidence intervals of change in FEV1 compared to post saline value shown.

GSK256066 significantly reduced the EAR (see Fig 3), inhibiting the fall in both minimum and weighted mean FEV1 by 40.9% (p = 0.014) and 57.2% (p = 0.014) respectively compared to placebo. GSK256066 also significantly reduced the LAR, attenuating the fall in both minimum and weighted mean FEV1 by 26.2% (p = 0.007) and 34.3% (p = 0.005) respectively compared to placebo.

Methacholine reactivity at 24 hrs post allergen challenge was not different after treatment with GSK256066 compared to placebo; the PC20 was 0.31 compared to 0.39 mg/mL respectively, geometric mean (95% CI) doubling dose difference -0.31 (-1.18 to 0.57).

### Pulmonary Function and FeNO

The FEV1 and FeNO measurements on day 7 at pre-dose and 1 hr post dose are shown in tables 2 and 3 respectively. There was no difference between the treatments for either of these measurements.

**Table 2:** Lung Function

	Day 1 Pre-dose	Day 7 Pre-dose	Day 7 1 hr post-dose
GSK256066 (L)	3.29 (2.88 to 3.69)	3.36 (2.94 to 3.79)	3.47 (3.04 to 3.90)

Placebo (L)	3.27 (2.85 to 3.69)	3.26 (2.91 to 3.60)	3.36 (2.99 to 3.73)

Adjusted treatment difference GSK256066 vs placebo (L)		0.09 (-0.03 to 0.20)	0.09 (-0.08 to 0.27)

Mean values shown, and with adjusted mean (95% confidence intervals) for treatment differences and ratios

**Table 3:** Exhaled Nitric Oxide

	Day 1 Pre-dose	Day 7 Pre-dose	Day 7 1 hr post-dose
GSK256066 (ppb)	39.9 (31.0 to 51.3)	34.7 (26.7 to 45.0)	36.1 (28.1 to 46.3)

Placebo (ppb)	34.5 (24.6 to 48.4)	33.1 (22.8 to 47.9)	34.3 (23.2 to 50.6)

Adjusted treatment ratio GSK256066 vs placebo		0.98 (0.85 to 1.31)	1.03 (0.88 to 1.20)

Mean values shown, and with adjusted mean (95% confidence intervals) for treatment differences and ratios

### Pharmacokinetics

**Figure 4:**
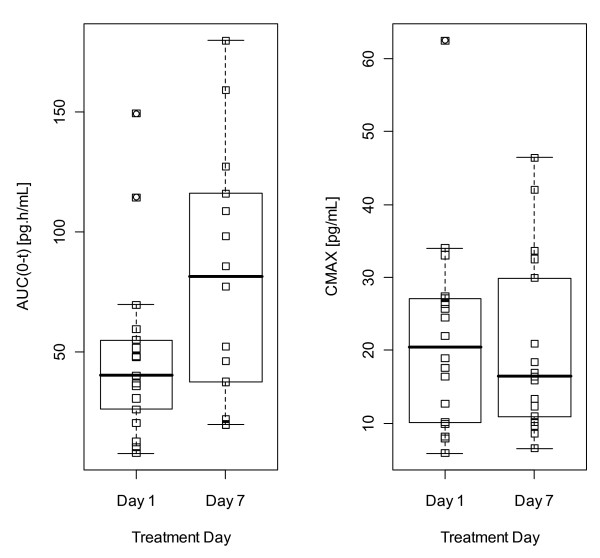
**Plot of individual derived plasma PK parameters AUC(0-t) and Cmax for GSK256066 on days 1 and 7**. Summary Box and Whisker plot overlaid. Open squares represent individual values. Central line, box and whisker limits represent median, interquartile range and most extreme value with 1.5× interquartile range, respectively.

The pharmacokinetic parameters for GSK256066 are shown in table 4, with individual data shown in Fig 4. GSK256066 concentrations were above the LLOQ in 18 out of 22 subjects on Day 1 and 17 out of 19 subjects on Day 7. Despite a very sensitive bioanalytical method (LLOQ 5 pg/mL), on day 1, the drug levels were below the LLOQ after 4 hrs post dose for the majority (18) of the 22 subjects. On day 7, 10 of the 19 subjects had drug levels were below the LLOQ after 4 hrs post dose. Only 1 subject on day 7 had levels above the LLOQ after 12 hrs. The systemic exposure to GSK614917 was also low, as only 8 out of 22 subjects had levels above the LLOQ on day 1, and 9 out of 19 on day 7. Variability in systemic exposure was high (coefficients of variation of AUC(0-t) and Cmax on day 1 of 89% and 68%, respectively), reflecting the difficulty in accurate characterisation of pharmacokinetic parameters when measurable concentrations are close to the limit of detection (Tlast ranged from 0.5-24 hours in this study).

**Table 4:** Pharmacokinetic Analysis for GSK256066.

Parameter	unit	Day 1	Day 7
AUC (0-t)	pg.h./mL	36.8 (93)	64.8 (89)

Cmax	Pg/mL	18.3 (68)	17.3 (63)

Tmax	h	1.0 (0.17-3.00)	1.0 (0.17-11.0)

Tlast	h	3.0 (0.5-6.0)	4.0 (1.0-24)

Values are geometric means (CV%) for AUC(0-t) and Cmax, and medians (range) for Tmax and Tlast. 0-t = From 0 hrs to time of last measurable concentration. N = 18 on Day 1, and n = 17 on Day 7.

## Discussion

To our knowledge, this is the first study to show that an inhaled PDE4 inhibitor inhibits the response to allergen challenge in asthma. This placebo controlled study demonstrated that GSK256066 administered for 7 days significantly attenuated the fall in lung function in patients with asthma caused by inhaled allergen challenge. GSK256066 had no effect on the secondary endpoints of methacholine reactivity post allergen challenge or exhaled nitric oxide. Nevertheless, the effects of GSK256066 on the allergen response which was the primary endpoint indicate that this drug has therapeutic potential for the treatment of asthma. The delivery of this PDE4 inhibitor by inhalation was associated with low systemic exposure. Larger clinical trials are needed to study the therapeutic index in more detail.

Inhaled allergen challenge is a well recognised and robust model that is commonly used to assess the therapeutic potential of novel treatments for asthma [11,12,19-24]. Comparing the results of different allergen challenge studies should be done with caution, as methodological details such as the period of measurement of the late response can vary between studies (we measured up to 10 hrs while some studies only measure up to 7 hrs), and individual patient characteristics may differ. The results of the current study are therefore not directly comparable to the previous publication involving the orally administered PDE4 inhibitor roflumilast, which inhibited the maximal fall in the EAR and LAR by 14% and 33% respectively. Inhibition of 40.9% and 26.2% respectively were observed in the current study. Direct head-to-head comparisons would be the best way to compare GSK256066 to roflumilast.

Inhaled corticosteroids attenuate the fall in lung function caused by inhaled allergen, with results varying between studies for the absolute magnitude of inhibition depending on the dose and type of corticosteroid used [20,22-24]. However, inhaled corticosteroids generally have a minimal effect on the EAR [20,22,23]. This may be due to the inability of corticosteroids to prevent mast cell degranulation. In contrast, GSK256066 had a very significant inhibitory effect on the EAR. PDE4 inhibition by GSK256066 may therefore offer more protection than corticosteroids against acute bronchoconstriction in clinical practice.

The LAR is characterised by an inflammatory cell influx into the airways, comprising a variety of cell types including eosinophils, basophils and lymphocytes that are recruited by T-helper 2 (TH2) cytokines [25]. The LAR is therefore a well validated model to study inhibition of TH2 driven inflammatory cell influx into the airways. Corticosteroids inhibit inflammatory gene transcription [26], and therefore decrease the number and activity of inflammatory cells at tissue sites of inflammation. Inhaled corticosteroids therefore inhibit airway inflammation during the LAR [20,22-24]. The leukotriene receptor antagonist montelukast also inhibits TH2 driven inflammation, and suppresses the LAR [19,20]. Similarly, it has recently been shown that targeting the TH2 cytokines IL-4 and IL-13 by blocking their common receptor with the IL-4 variant pitrakinra also inhibits the LAR [21]. PDE4 is expressed on cells involved in TH2 responses, such as eosinophils and lymphocytes [5,27]. The current study would have been strengthened by proving that GSK256066 had an effect on these TH2 cells. Nevertheless, our results agree with previous findings using roflumilast showing that PDE4 inhibition attenuates the LAR [12], suggestive of inhibition of TH2 inflammation.

There was no change in the secondary endpoint measurements of methacholine challenge post allergen, or exhaled NO. However, the study was not statistically powered to examine these secondary endpoints, but was designed to evaluate the primary endpoint of the allergen challenge, where unequivocally positive results were observed. Studies using inhaled corticosteroids have shown both attenuation [20,24] and no attenuation [22] of methacholine reactivity post allergen challenge. In line with these variable results, montelukast has also been shown to have no effect on methacholine reactivity post allergen challenge in one study [20] but an inhibitory effect in another [19]. These variable results suggest that methacholine reactivity post allergen challenge is not a robust primary endpoint to evaluate drug effects. It is clear that GSK256066 inhibits the fall in lung function during the LAR, but unlike corticosteroids [20,24] we did not observe inhibition of allergen induced bronchial hyper-reactivity. This may suggest differentiation of the effects of PDE4 inhibitors and corticosteroids, although the inconsistent results in previous studies of methacholine reactivity post allergen challenge indicate that caution should be applied in the interpretation of these data.

Reducing nitric oxide levels by specific iNOS inhibition does inhibit the EAR or LAR, suggesting that nitric oxide is not mechanistically involved in the pathophysiology of asthma [19]. However, exhaled nitric oxide is a sensitive biomarker of the effects of inhaled corticosteroids [28]. In contrast, the effects of the leukotriene receptor antagonist singulair are more variable, with no inhibition observed of nitric oxide observed in some studies [19,29]. The usefulness of exhaled nitric oxide as a biomarker appears to vary with the class of drug, and our results suggest that airway nitric oxide production is a PDE4 independent mechanism. Alternative explanations are that the current study was too short or underpowered to detect a reduction in exhaled nitric oxide.

There were few adverse effects in this study, although larger studies are needed to fully explore the safety profile. However, the lack of nausea and/or gastro-intestinal side effects usually associated with oral PDE4 inhibitors [15-17] indicates that the inhaled delivery of a PDE4 inhibitor may minimise the potential for systemic side effects. The pharmacokinetic analysis performed showed that systemic exposure to GSK256066 was extremely low, as some subjects did not have quantifiable exposure at any time-point despite measurement with a very sensitive analytical assay (LLOQ of 5 pg/mL). Furthermore, the majority of subjects had levels below the LLOQ after 4 hrs on days 1 and 7. Additionally, the mean Cmax of GSK256066 was <20 pg/ml on both of these days, while measurable levels of the active metabolite GSK614917 were even lower, underscoring the value of inhaled delivery to limit systemic exposure and the potential for systemic side effects. In contrast, the mean Cmax of roflumilast administered orally is over 2,000 pg/ml with levels of the active metabolite roflumilast N-Oxide being even higher [30]. Clearly orally administered drugs will have higher plasma levels, but this comparison serves to highlight the low levels of systemic exposure with inhaled delivery for GSK256066.

Two subjects were withdrawn from this study with high creatinine clearance values. This is because the protocol stated that subjects with abnormal creatinine clearance values defined by the laboratory reference range should be withdrawn, in order to exclude patients who developed renal dysfunction. High creatinine clearance indicates good renal function, so there was no clinical concern about keeping these patients in the study. However, the wording of the protocol stated that we had to withdraw these patients as the values were outside the laboratory reference range. In retrospect, the protocol should have stated that patients with low creatinine clearance would be withdrawn.

It has recently been reported that the inhaled PDE4 inhibitor UK-500,001 had no effect on FEV1 after 6 weeks of treatment in patients with COPD [31]. Oral PDE4 inhibitors have been reported to show clinical efficacy in COPD patients [15-17], but with a significant rate of side effects. The effects of PDE4 inhibitors will therefore vary according to a variety of factors including the potency of the drug and the route of delivery. The current study using inhaled GSK256066 was focused on asthma, and studies using this drug in COPD would be of interest.

This was the first time that GSK256066 had been given to patients with asthma, and so the side effect profile in this population was unknown. PDE4 inhibitors are known to cause adverse effects [15-17], so we wanted to limit the duration of exposure in case GSK256066 caused significant adverse effects. We chose 7 days treatment in order to limit the duration of exposure to a new drug with an unknown side effect profile, while at the same time treating for long enough to be able to measure any therapeutic effect. Future studies can use the preliminary safety data from the current study to investigate safety and efficacy over a longer duration, or using other dosing regimens.

In summary, we show that the inhaled PDE4 inhibitor GSK256066 attenuates the allergen induced changes in pulmonary function in asthmatics. By limiting systemic exposure, this therapy has the potential to minimise side effects usually associated with PDE inhibitors, and warrants further study in longer clinical trials.

## Abbreviations

PDE: Phosphodiesterase; cAMP: adenosine monophosphate; cGMP: cyclic guanosine monophosphate; FEV_1_: Forced expiratory volume in 1 second; FeNO: Exhaled nitric oxide; EAR: Early asthmatic response; LAR: Late asthmatic response; PC_20_: Provocative concentration to reduce the FEV_1 _by 20%.

## Competing interests

DS works on a consultancy basis for GSK, Chiesi Pharmaceuticals, AstraZeneca, CIPLA and Allmiral

BO has had ad-hoc consultancy arrangements with several of the major pharmaceutical companies involved in research and marketing of therapeutic agents for respiratory disease. These include GlaxoSmithKline, AstraZeneca, Altana, Aventis, Celgene, Pfizer, Boehringer Ingelheim and various small biotechnology companies. As consultant to these companies he serves on advisory boards to provide expert input into development of new products, clinical trial design, development of protocols and slide presentations. The honorarium he receives for these consultancies ranges for $600-1,200. He has never received more than $3,000 in any one year from any company. He does not and never has had any stock or other equity ownership in pharmaceutical or biotechnology companies. He does not at present and never has had a patent licensing arrangement with any company. He receives grant and research support from several pharmaceutical companies. He directs a phase 2 clinical research unit, dedicated to the evaluation of new drugs for airways disease. As a result, he performs a number of studies, similar to that reported in this manuscript. He does not personally benefit financially from any grant or research support income. He has spoken for AstraZeneca, GSK, Pfizer, Boehringer Ingelheim and Altana for several years. The speakers honorarium never exceeds $1,000. His annual speaker fees never exceeds a total of $10,000. He has provided this statement based on his recollection of activities in partnership with pharmaceutical companies and other commercial associations that might be considered to pose a conflict of interest. He does not believe that his relationship with these organisations presents a conflict of interest to his authorship of this manuscript.

AJM and ALL are GSK employees.

FP was a GSK employee, and now works for AMGEN.

## Authors' contributions

DS was involved in study design, patient recruitment, organisation of study conduct, interpretation of results and drafted the paper.  FP was involved in study design and analysis of the data.  AJM was involved in study design, and was the lead for analysis and interpretation of the pharmacokinetic data.  AL was involved in study design and interpretation of the results.  BOC was involved in study design, patient recruitment, organisation of study conduct, interpretation of results and writing of the paper. All authors have read and approved the final manuscript.
